# Allergen Challenge Induces Ifng Dependent GTPases in the Lungs as Part of a Th1 Transcriptome Response in a Murine Model of Allergic Asthma

**DOI:** 10.1371/journal.pone.0008172

**Published:** 2009-12-21

**Authors:** Nilesh Dharajiya, Swapnil Vaidya, Mala Sinha, Bruce Luxon, Istvan Boldogh, Sanjiv Sur

**Affiliations:** 1 Department of Internal Medicine, University of Texas Medical Branch, Galveston, Texas, United States of America; 2 Department of Biochemistry and Molecular Biology, University of Texas Medical Branch, Galveston, Texas, United States of America; 3 Department of Microbiology, University of Texas Medical Branch, Galveston, Texas, United States of America; New York University, United States of America

## Abstract

According to the current paradigm, allergic airway inflammation is mediated by Th2 cytokines and pro-inflammatory chemokines. Since allergic inflammation is self-limited, we hypothesized that allergen challenge simultaneously induces anti-inflammatory genes to counter-balance the effects of Th2 cytokines and chemokines. To identify these putative anti-inflammatory genes, we compared the gene expression profile in the lungs of ragweed-sensitized mice four hours after challenge with either PBS or ragweed extract (RWE) using a micro-array platform. Consistent with our hypothesis, RWE challenge concurrently upregulated Th1-associated early target genes of the Il12/Stat4 pathway, such as p47 and p65 GTPases (Iigp, Tgtp and Gbp1), Socs1, Cxcl9, Cxcl10 and Gadd45g with the Th2 genes Il4, Il5, Ccl2 and Ccl7. These Th1-associated genes remain upregulated longer than the Th2 genes. Augmentation of the local Th1 milieu by administration of Il12 or CpG prior to RWE challenge further upregulated these Th1 genes. Abolition of the Th1 response by disrupting the Ifng gene increased allergic airway inflammation and abrogated RWE challenge-induced upregulation of GTPases, Cxcl9, Cxcl10 and Socs1, but not Gadd45g. Our data demonstrate that allergen challenge induces two sets of Th1-associated genes in the lungs: 1) Ifng-dependent genes such as p47 and p65 GTPases, Socs1, Cxcl9 and Cxcl10 and 2) Ifng-independent Th1-inducing genes like Gadd45g. We propose that allergen-induced airway inflammation is regulated by simultaneous upregulation of Th1 and Th2 genes, and that persistent unopposed upregulation of Th1 genes resolves allergic inflammation.

## Introduction

Allergic asthma is characterized by airway eosinophilia, bronchial hyperresponsiveness, increased mucin production, and allergen-specific IgE response. Current evidence implicates Th2 cytokines and chemokines like Il4, Il5, Il13, Ccl2 and Ccl7 in the pathogenesis of allergic inflammation. Il4 is the principal cytokine that stimulates IgE class-switching in B cells and directs Th2 helper cell development from Th0 cells [1], [2]. Mice deficient in the Il4 gene have low IgE levels, deficiency in the development and maintenance of Th2 cells, and an attenuated allergic airway inflammation in murine models [3], [4]. The major role of Il5 is to stimulate differentiation of eosinophil precursors and activation of mature eosinophils [5]. Il13 appears to be mainly responsible for producing biological effects of allergic inflammation by stimulating mucus production, inducing endothelial cell expression of adhesion molecules and chemokines, promoting fibrosis and stimulating airway smooth muscle contractility [6]. We and others have previously reported the role of the pro-inflammatory chemokines Ccl2 and Ccl7 in allergic airway inflammation [7], [8].

Allergic airway inflammation is self-limiting, and resolves after the offending allergen is removed. The Th1 cytokine Ifng regulates allergic airway inflammation, and may inhibit Th2 differentiation by Tbet-mediated suppression of Gata3 [9], [10]. Similarly, the Th1-inducing cytokine Il12 and CpG oligonucleotide can inhibit Th2 differentiation [11]. A number of Th1- and Ifng-associated genes have been identified. Gadd45g has been reported to be a Th1-skewing gene that is induced during T cell activation and is expressed at higher levels in Th1 than in Th2 cells [12]. Socs1, a member of the SOCS (suppressor of cytokine signaling) family of proteins characterized by the presence of a Src homology 2 domain and a C-terminal conserved domain called the SOCS box, has been proposed as Th1 lineage marker [13]. Iigp and Tgtp are members of a family of Ifng-induced 47 kD GTPases [14]. Whether these genes play a role in regulating allergic inflammation is not known.

Gene micro-arrays have been used to characterize gene expression profiles in animal models of allergic airway inflammation [14]–[16]. These studies demonstrated that inhalation of allergens in sensitized animals vigorously induces pro-Th2 cytokine and chemokine genes such as Il4, Ccl2, Ccl7 and Ccl11 [15]. Thus use of micro-arrays appears to be a valid high-throughput approach to assess gene expression patterns in allergic disorders. However, no studies to date have looked at global gene expression profiles to identify genes that might regulate and resolve allergic inflammation.

Since allergic inflammation is self-limited, we hypothesized that allergen challenge simultaneously induces anti-inflammatory genes to counter-balance the effects of Th2 cytokine and chemokine genes. To identify potential regulatory genes, we utilized gene microarrays to examine gene expression profiles in the lungs of sensitized mice challenged with RWE.

## Materials and Methods

### Ethics Statement

All animal experiments were approved by the Institutional Animal Care and Use Committee of the University of Texas Medical Branch.

### Mice

Six to eight week-old female wild-type (WT) and Ifng knock-out (KO) BALB/c mice were purchased from Jackson Laboratories (Bar Harbor, Maine). All mice were maintained in a specific pathogen-free environment throughout the experiment.

### Ragweed

We have previously shown that endotoxin contamination alters inflammatory cell recruitment after allergen challenge [17]. To avoid this problem, endotoxin-free ragweed (lot XP56-D10-1320) was purchased from Greer Laboratories (Lenoir, NC).

### Model of Allergic Sensitization and Challenge

WT and Ifng KO Balb/c mice were sensitized by two intraperitoneal (i.p.) injections of endotoxin-free RWE (150 µg) and alum on days 0 and 4. On day 11, allergen challenge was performed by intranasal (i.n.) instillation of RWE (200 µg) in anesthetized mice. Mice were sacrificed at various time points as indicated after the challenge, and bronchoalveolar lavage (BAL) fluid and lungs were collected. Mice sensitized but not challenged served as the 0 hour time point. For the Th1/CpG experiments, mice were treated intranasally with CpG DNA (35 µg/mouse) or Il12 (10 ng/mouse) 48 hours and 16 hours before RWE challenge, respectively. Lungs were harvested 4 hours later.

### Quantitative RT-PCR Analysis

Immediately after the mice were sacrificed, lungs were removed, frozen rapidly in liquid nitrogen, and stored at −80°C until RNA extraction. RNA was extracted and quantitative PCR analyses were performed using the SYBR green real-time PCR kit (Applied Biosystems) as described previously [18]. Gene-specific primer pairs used are shown in Table 1.

**Table 1:** Primers used in qPCR experiments.

Gene	Forward Primer	Reverse Primer
Il4	AGATCATCGGCATTTTGAACG	TTTGGCACATCCATCTCCG
Il5	TGCCTGGAGCAGCTGGAT	GTGGCTGGCTCTCATTCACA
Ccl2	CCCACTCACCTGCTGCTACT	CAGCTTCTTTGGGACACCTG
Ccl7	CCCAAGAGGAATCTCAAGAGC	GACTTCCATGCCCTTCTTTG
Fos	GCCAGTCAAGAGCATCAGCA	ACAGCTTGGGAAGGAGTCAG
Iigp	CTGGGATTGGAAGCACAAAT	AATCCTTCCAGCCAAATCCT
Gbp1	CCCTGTATGGAGAACGCAGT	GGGCATGTGGATCTTCTGAT
Spcs1	AGTAGGATGGTAGCACG	TCAGGTAGTCACGGAGT
Gadd45g	CCGTGGCCAGGATACAGTTC	TCGTTGAAGCTGCGGCTCTC
Ifng	AGCTCATCCGAGTGGTCCAC	AGCAGCGACTCCTTTTCCG
β-actin	CCTTCAACACCCAGCCATGT	TGTGGACCACCAGAGGCATAC
Cxcl9	CAGAACCTCCCACGTAGCTTTC	GCTCTGAAGATGGGATCAAGTTAATA
Cxcl10	CTAGTCCTAATTGCCCTTGGTCTT	AGGAGGAGTAGCAGCTGATGTGA

### Gene Microarrays

Microarray data are Minimum Information About a Microarray Experiment (MIAME)-compliant, and the raw data have been deposited in the Gene Expression Omnibus (GEO), a MIAME-compliant database (Accession number- GSE18083). The analysis was done using Mouse Genome 430_2.0 Affymetrix chips containing 14,000 well- characterized genes. The experimental procedures were performed according to the manufacturer's instructions. Briefly, double-stranded cDNA was prepared from 10 µg of total lung RNA using the SuperScript Choice kit (Invitrogen, Carlsbad, CA). Purified cDNA (10 µl) was used in an *in vitro* transcription reaction with the Bioarray High Yield RNA Transcript labeling kit, according to the manufacturer's protocol (Enzo, Farmingdale, NY). Biotin-labeled antisense cRNA was purified with the use of an RNeasy mini-kit as suggested by the manufacturer (Qiagen, Hidden, Germany). The cRNA yield was determined by measuring UV absorbtion at 260 nm. To improve the hybridization efficiency, 15 µg of cRNA was fragmented and used to create a GeneChip hybridization solution as suggested by the manufacturer (Affymetrix, Santa Clara, CA). In addition, aliquots of a solution of eleven bacterial RNA transcripts, spanning a range of known frequencies, were “spiked” into each hybridization solution as an internal normalization control. Hybridization solutions were pre-hybridized to two glass beads (Fisher Scientific, Pittsburgh, PA) at 45°C overnight. The hybridization solution was removed to a clean tube and heated for 1–2 min at 95°C and microcentrifuged on high for 2 minutes to pellet insoluble debris. These solutions were then hybridized to the gene chips. Gene chips were hybridized with 180 µl of the hybridization solution at 45°C and rotating at 45–60 rpm overnight. After overnight incubation the hybridization solutions were recovered and chips were washed and prepared for scanning per the manufacturer's protocols. Raw fluorescence data were collected and analyzed with the use of the GeneChip 3.1 application (Affymetrix, Santa Clara, CA) to create the primary data, i.e., the calculated gene frequencies.

### Analyses of Changes in Gene Expression

Microarray experiments were performed according to the manufacturer's instructions in accordance with Minimum Information About a Microarray Experiment (MIAME) guidelines. Student's t-test was done on the RMA + Quantile summarized data using S+ Array Analyzer software (TIBCO, Palo Alto, CA). The t-test way was performed on these genes, which at a p-value <0.01 identified 352 genes that were differentially expressed between the PBS and RWE challenge groups. These genes were further analyzed using Spotfire™ (TIBCO Software) and the Ingenuity Pathway Analysis knowledge database (Ingenuity Systems, Redwood City, CA).

### BAL and Cell Count

Mice were anesthetized with an i.p. injection of ketamine and xylazine. A tracheotomy was performed and the trachea was cannulated. BAL of both lungs was performed twice with 0.7 ml of sterile PBS (pH 7.3) through the tracheal cannula with a syringe. The BAL was centrifuged at 4°C for 10 min at 400×*g*, and the pellet was suspended in 750 µl of ice-cold Dulbeco's PBS (Sigma-Aldrich, St. Louis, MO). Total cell counts in BAL were determined from an aliquot of the cell suspension. Differential cell counts were done on cytocentrifuge preparations (Cytospin 3; Thermo Shandon, Pittsburgh, PA) stained with Wright-Giemsa, counting 200 cells from each animal.

## Results

### Allergen-Induced Th2 Gene Upregulation in the Lungs Precedes Eosinophil Recruitment

**Figure 1:**
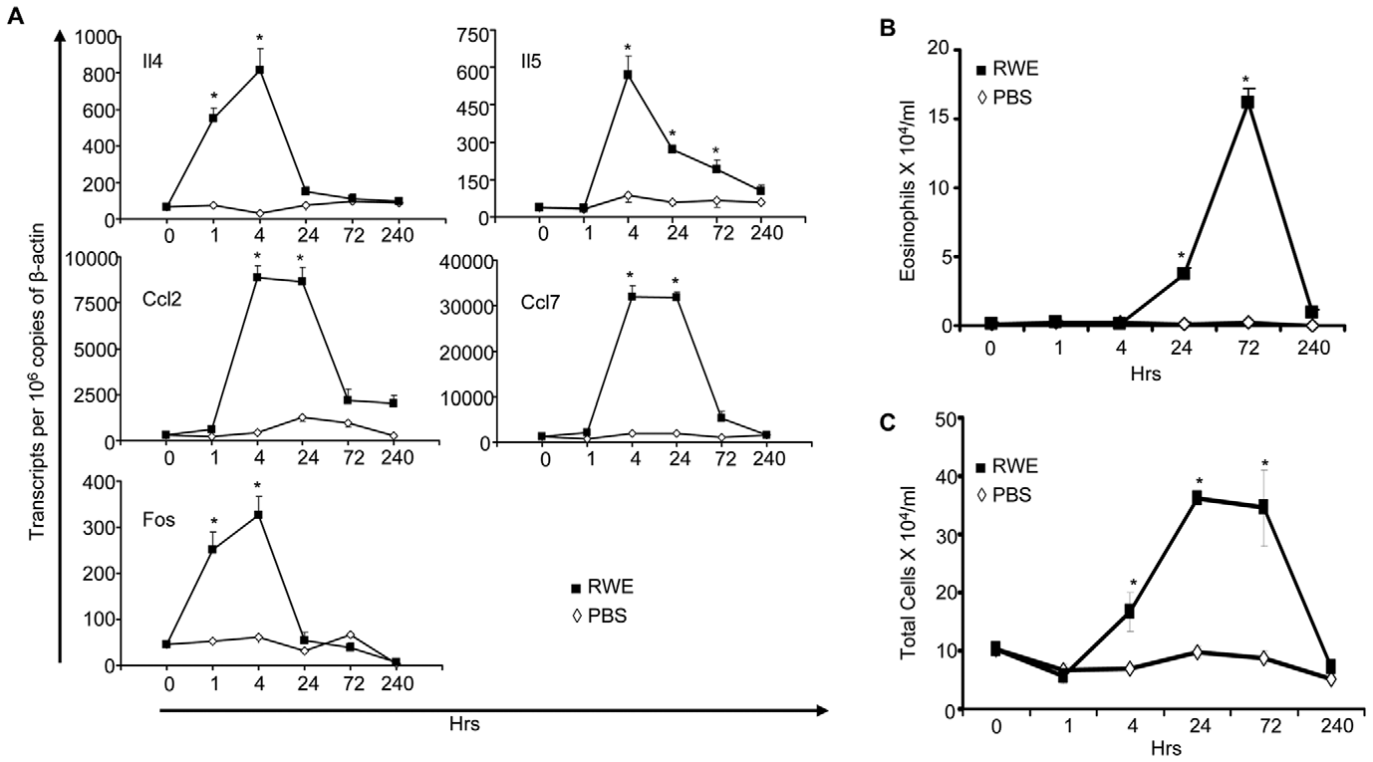
Kinetics of Th2 gene expression and eosinophil recruitment after RWE challenge. (**A**) Wild-type BALB/c mice (n = 5−8 per time point) were sensitized with RWE and challenged with either RWE (▪) or PBS (◊) as described. Total RNA was extracted from lungs collected at 1, 4, 24, 72 and 240 hrs after the challenge and subjected to real-time quantitative PCR analyses using gene-specific primers. Transcript abundance is presented here as a function of time, after normalizing to 10^6^ β-actin transcripts. (**B**) and (**C**) BALB/c mice (n = 5−8 per time point) were sensitized with RWE and challenged with either RWE (▪) or PBS (◊) as described. BAL was performed at 1, 4, 24, 72 and 240 hrs after the challenge. BAL eosinophil (B) and total cell (C) counts were determined using Wright-Geimsa-stained cytospin slides. Data are represented as mean±SEM; _*_ = P≤0.05.

We have previously reported that the protocol for sensitization and challenge with ragweed used in the current study induces allergen-specific IgE, eosinophilic airway inflammation and airway hyper-responsiveness. [11], [19]–[22]. We performed kinetic analysis of Th2 gene upregulation and allergic airway inflammation after RWE challenge in sensitized mice. Compared to PBS, RWE challenge upregulated Th2 cytokine (Il4 and Il5) gene expression that peaked at four hours post- challenge and returned to baseline by 1–10 days (**Figure 1A**). Il4 and Il5 mRNA expression increased 12-fold and 15-fold, respectively. At four hours, RWE challenge also maximally upregulated C-fos, Ccl2 and Ccl7 by 27- to 30-fold (**Figure 1A**). These findings demonstrate that Th2-associated genes peak at 4 hours post-allergen challenge. Kinetic analysis of airway inflammation showed that RWE challenge did not induce detectable eosinophil recruitment at this time point. Eosinophil recruitment began 24 hours after challenge, peaking at 72 hours, with almost complete resolution by day 10 (**Figure 1B**). Ragweed challenge induced an increase in the number of total inflammatory cells, starting at four hours and peaking at 72 hours (**Figure 1C**). Similarly, lymphocyte, macrophage and neutrophil recruitment began at 4–24 hours after challenge and peaked at 24–72 hours (data not shown). These observations indicate that RWE challenge upregulates Th2 genes before eosinophil recruitment in the lungs.

### RWE Challenge in Sensitized Mice Induces Ifng-Dependent GTPases and Th1-Associated Genes in the Lungs

**Figure 2:**
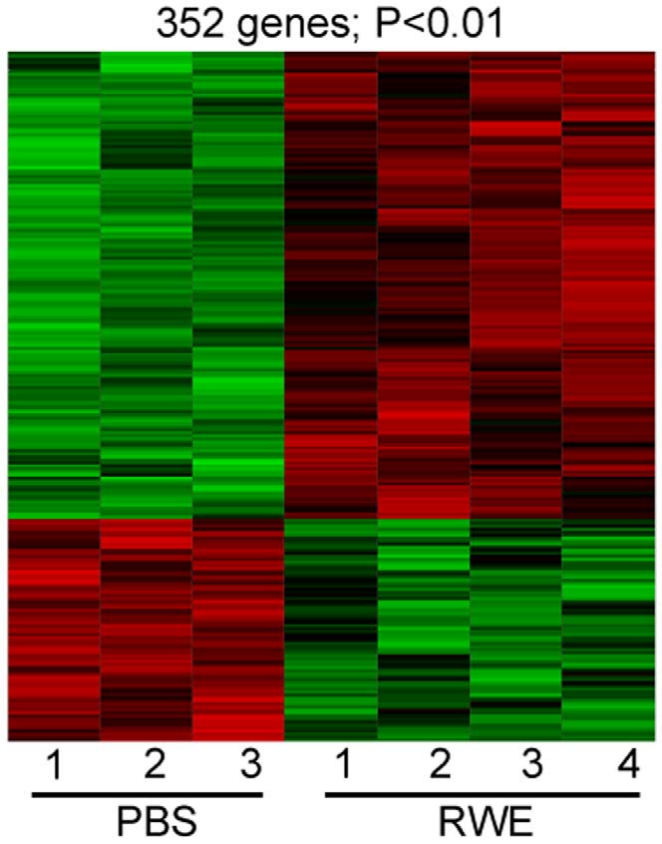
Heat map of genes significantly altered in the lungs by RWE challenge. Sensitized Balb/c mice were challenged with either PBS (n = 3) or RWE (n = 4) and gene micro-array was performed 4 hours post-challenge. Student's t-test was performed on the raw data from the micro-array analysis using the S+ Array Analyzer software. This analysis, at a P<0.01, identified 352 genes that were differentially expressed between the PBS and RWE challenge groups. These 352 genes were analyzed using Spotfire to generate this heat map. The numbers below the lanes represent data from individual mice.

**Figure 3:**
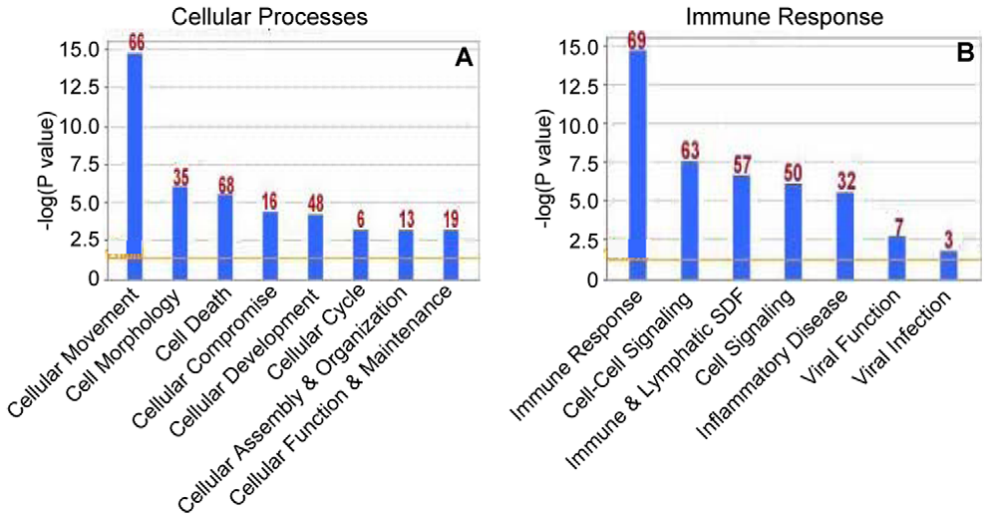
Processes, functions and pathways altered by RWE challenge. Genes altered by RWE challenge were analyzed using the Ingenuity Pathway Analysis (IPA) knowledge database. Cellular Processes (A) and Immune Response (B) were the top two processes affected. Bar graphs show functions/pathways within each process significantly altered by RWE challenge (P<0.05). The Y-axis denotes the significance of altering that particular function; a higher bar means greater significance. The yellow line is the cut-off for the significance level and coincides with a P value of 0.05. The number above each bar denotes the number of genes in that particular function that were significantlyaltered by RWE challenge (P<0.01).

Eosinophil recruitment to the lungs is likely to be regulated by pro- and anti-inflammatory genes whose expression peak before the influx of this cell type. Building on our observation that Th2 gene upregulation precedes eosinophil recruitment, we hypothesized that a set of anti-inflammatory genes must be simultaneously upregulated in the lungs. Since Th2 gene expression peaked at fours hours after allergen challenge, we performed our micro-array experiments at this time point to identify anti-inflammatory genes. Since endotoxin can induce Th1-associated genes, we used endotoxin-free RWE to prevent confounding of the results [17]. Gene expression in the lungs of PBS (n = 3)- and RWE (n = 4)-challenged mice was analyzed using Affymetrix Mouse Genome 430 2.0 chips. RWE challenge altered (increased or decreased) the expression of 352 genes in the lungs (P<0.01). A heat map of the gene profiles is shown in **Figure 2**. Consistent with the qPCR results above (**Figure 1A**), the gene array detected upregulation of Il4, Il5, Ccl2 and Ccl7. We used the Ingenuity Pathways Analysis (IPA) knowledge base to identify the functional categories to which these 352 genes belong. **Table 2** shows the biological processes and number of genes within each process significantly altered by RWE challenge. Further analysis of the top two processes (Cellular Processes and Immune Response) is shown in **Figure 3**. As expected in this model of allergic airway inflammation, many of the known Th2-associated genes were upregulated after RWE challenge (**Table 3**). Consistent with our hypothesis, RWE challenge simultaneously induced Iigp, Tgtp, Socs1, Gadd45g and Cxcl10 in the lungs, genes that have previously been described as Ifng- or Th1-dependant [12], [13], [23] (**Table 4**).

**Table 2:** Biological processes altered by RWE challenge.

Process	No. of genes altered within the process
Cellular Processes	130
Immune Response	96
Cellular Growth & Proliferation	69
Modification at DNA, RNA and Protein Levels	53
Molecular Transport	43
Small Molecule Biochemistry	39
Organismal Injury and Abnormalities	20
Amino Acid Metabolism	14

Genes altered by RWE challenge were analyzed using the Ingenuity Pathway Analysis knowledge database and grouped into biological processes as shown.

**Table 3:** Th2-associated genes upregulated by RWE challenge.

Gene	Accession number	Fold change with RWE challenge
Il4	NM_021283	4.2
Il5	NM_010558	1.3
Il10	NM_010548	1.4
Il4ra	NM_010557	1.5
Ccl2	AF065933	2.2
Ccl7	AF128193	2.9
Ccl11	NM_011330	5.9
Il1r1	NM_008362	1.3
Ccr2	BB148128	1.5
Icos	AB023132	1.4

Fold change analysis was done using the S+ Array Analyzer software.

**Table 4:** Th1/Ifng-associated genes upregulated by RWE challenge.

Gene	Accession number	Fold change with RWE challenge
Iigp	BM239828	2.9
Tgtp	NM_011579	1.4
Socs1	AB000710	3.0
Gadd45g	AK007410	1.8
Cxcl10	NM_021274	2.7

Fold change analysis was done using the S+ Array Analyzer software.

### RWE Challenge Simultaneously Upregulates Th1- and Th2-Associated Genes

**Figure 4:**
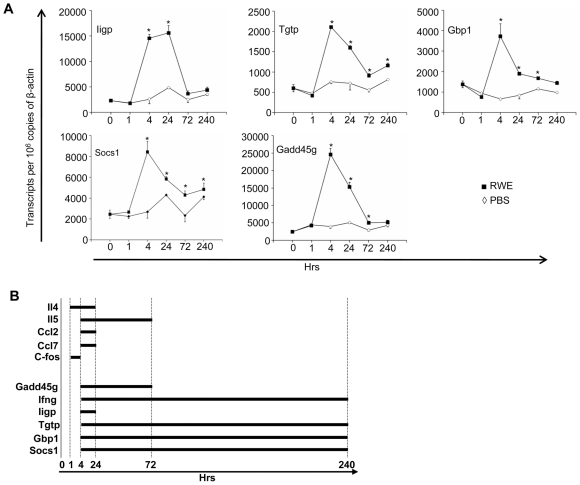
RWE-induced upregulation of GTPases, Socs1 and Gadd45g. (**A**) Sensitized Balb/c mice were challenged with either PBS (◊) or RWE (▪). Lungs were harvested from mice sacrificed at different time points. Total RNA was extracted from the lungs and subjected to real-time quantitative PCR analyses using gene-specific primers. Transcript abundance is presented here as a function of time after normalizing to 10^6^ β-actin transcripts. Data are represented as mean±SEM; * = P<0.05. (**B**) Schematic representation of the time frame for which the Th2 genes (upper group) and Th1 genes (lower group) remain upregulated after RWE challenge.

We confirmed the induction of these Th1-associated genes in the lungs and determined their kinetics by qPCR analysis. Consistent with the gene array results, RWE challenge upregulated the p47 GTPases, Iigp and Tgtp that peaked with a four- to seven-fold increase at 4–24 hours post-challenge (**Figure 4A**). An earlier gene-array study showed expression of the p65 GTPase Gbp1 in Th1-polarized CD4 T cells in addition to Iigp and Tgtp [12], [13], [23]. Hence, we also looked at the expression of Gbp1 and found it to be upregulated by three-fold upon RWE challenge. Likewise, RWE challenge also upregulated Socs1 and Gadd45g at four hours, with peak levels three-fold and 10-fold compared to baseline (**Figure 4A**). The upregulation of Tgtp, Gbp1 and Socs1 was sustained for 10 days (**Figure 4A**). Collectively, these data support our hypothesis that allergen challenge simultaneously induces Th2 and Th1 genes, but Th1 genes remain upregulated longer than Th2 genes (**Figure 4B**).

### Administration of the Th1-Inducing Agents Il12 or CpG DNA Augments RWE Challenge-Induced Upregulation of Th1-Associated Genes in the Lungs

**Figure 5:**
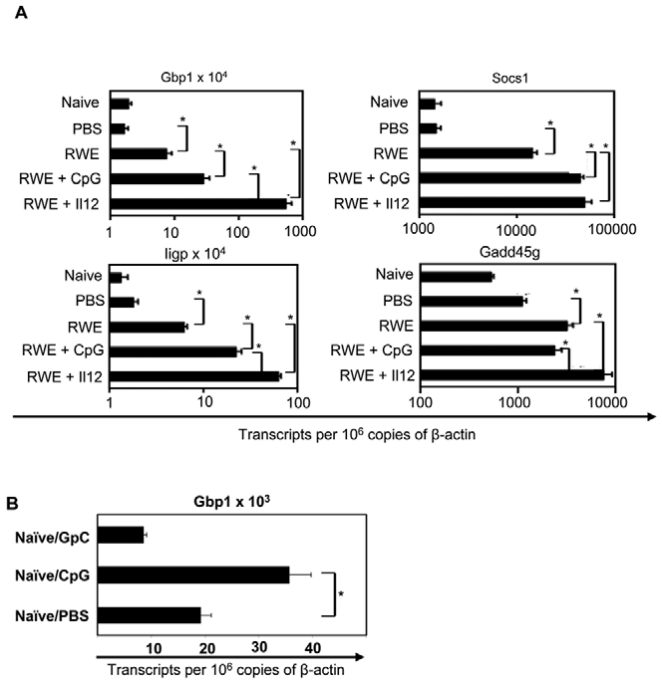
A Th1-differentiating environment augments upregulation of Iigp, Gbp1, Gadd45g and Socs1. (**A**) Sensitized Balb/c mice were challenged with either PBS or RWE. To generate Th1- differentiating conditions, mice were pre-treated intranasally with either Il12 or CpG DNA 16 or 48 hrs before RWE challenge, respectively. Mice were sacrificed 4 hrs after challenge and quantitative PCR analysis was performed and analyzed as described for Fig. 1A. (**B**) Naïve Balb/c mice were treated intranasally with PBS, CpG or GpC DNA. Mice were sacrificed 48 hours later and quantitative PCR was performed on the lungs. Data are represented as mean±SEM; * = P<0.05.

In previous studies we have demonstrated that administration of Th1-inducing Il12 or CpG DNA either before or during allergen challenge can inhibit allergic inflammation and provide long-term protection against allergic airway inflammation [24], [25]. We reasoned that if Ifng- and Th1- associated genes induced by RWE challenge provide protection against allergic inflammation, they are likely to be further upregulated by prior administration of Il12 or CpG DNA. Administration of Il12 and CpG DNA intranasally 16 and 48 hrs before RWE challenge, respectively, upregulated Gbp1, Iigp and Socs1 more than RWE challenge alone (**Figure 5A**). Similarly, Il12 but not CpG augmented Gadd45g transcripts in the lungs (**Figure 5A**). Intra-nasal administration of CpG DNA also upregulated Gbp1 in RWE-naïve mice (**Figure 5B**). These findings suggest that RWE challenge induces Gbp1, Iigp, Gadd45g and Socs1, consistent with a Th1 environment, and that Gbp1 upregulation was inducible by the Tlr9 pathway.

### Disruption of Ifng Abrogates RWE Challenge-Induced Upregulation of Iigp, Tgtp, Gbp1, Socs1 and Th1 Chemokines, Cxcl9 and Cxcl10

**Figure 6:**
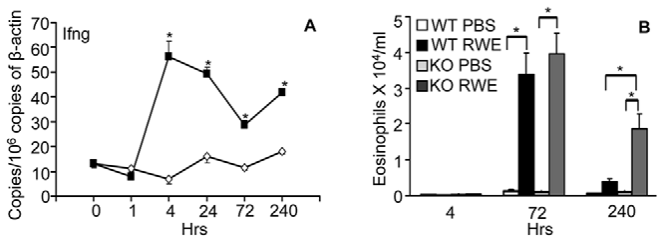
Upregulation and role of Ifng in RWE-induced allergic inflammation. (**A**) Sensitized Balb/c mice were challenged with either PBS (◊) or RWE (▪). Quantitative PCR was done on lung RNA four hours after challenge. (**B**) Sensitized WT or Ifng KO mice were challenged with either PBS or RWE. BAL was performed at different time points and eosinophils counted on Wright-Giemsa-stained cytospin slides. Data are represented as mean±SEM; * = P<0.05.

**Figure 7:**
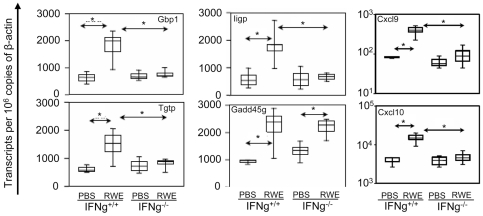
RWE-induced upregulation of GTPases, Cxcl9 and Cxcl10 is abrogated in Ifng KO mice. Sensitized Ifng^+/+^ and Ifng^−/−^ KO Balb/c mice were challenged with either PBS or RWE. Mice were sacrificed 4 hrs post-challenge, total RNA was extracted from the collected lungs, and quantitative PCR was performed. * = P<0.05.

The effects of Il12 and CpG DNA are mediated through Ifng [24], [26]. Building on our observation that IL12 and CpG DNA further upregulated RWE-challenge induced expression of Ifng and Th1-associated genes, we examined the upregulation of Ifng by RWE challenge. Compared to PBS, challenge with RWE upregulated Ifng, which peaked at 4 hours and was sustained for at least 10 days (**Figure 6A**). Next we looked at the role of Ifng in RWE-induced allergic inflammation using Ifng knockout mice. As expected, RWE challenge induced eosinophil recruitment in the airways at 72 hrs in WT and KO mice (**Figure 6B**). Compared to WT mice, airway eosinophilia induced by RWE challenge persisted at day 10 in Ifng KO mice (**Figure 6B**), indicating a critical role of Ifng in regulating allergic airway inflammation. Disruption of the Ifng gene abrogated RWE-induced upregulation of Gbp1, Iigp and Tgtp, but not Gadd45g (**Figure 7**). The chemokines Cxcl9 and Cxcl10 were likewise upregulated in response to RWE challenge in WT but not in Ifng KO mice (**Figure 7**). Together these findings suggest that Ifng and its downstream molecules like the p47/p65 GTPases, Cxcl9 and Cxcl10 may play a role in regulating eosinophilic airway inflammation.

## Discussion

Allergic airway inflammation is an IgE-mediated response associated with vigorous eosinophil recruitment to the lungs. Many studies have demonstrated a role for Th2 cytokines and pro-inflammatory chemokines in induction of allergic airway inflammation. The “global transcriptome” induced in the lungs by RWE challenge demonstrated upregulation of Ifng- and Th1-associated genes such as Iigp, Tgtp, Gbp1, Sosc1, Cxcl9 and Cxcl10 prior to eosinophil recruitment. This raises the question of why RWE challenge induced eosinophilic inflammation despite simultaneous induction of both Th2 and Th1 genes. In the early stages of gene induction RWE challenge upregulated Th2 genes by 21-fold on average, and Th1 genes by 5-fold. This dominant induction of Th2 genes over Th1 genes may explain the resultant Th2 inflammation.

The Th1-associated genes described in the present study have also been previously reported to be induced by Il12 signaling through Stat4 via induction of optimal levels of Ifng production and commitment of Th1 cells [*13*], [*14*], [*23*], [*27*]. Consistent with these reports, we demonstrate in our study that the Th1-promoting agents Il12 and CpG DNA further upregulate these genes, and that disruption of the Ifng gene inhibits RWE challenge-induced upregulation of these genes, while boosting allergic inflammation. Our observations are consistent with previous studies that suggest increased levels of the Th1 cytokine Ifng in patients with asthma exacerbation, and increased secretion of Ifng from BAL leukocytes of asthmatic patients [28], [29]. A recent study showed upregulation of the NDFIP2 gene during early Th2 differentiation in response to Il4 treatment [30]. NDFIP2 knockdown by shRNA decreased Ifng production by Th1 cells, suggesting that NDFIP2 promotes Ifng and Th1 differentiation [30]. Thus, Il4-induced Th2 differentiation also induces genes that promote Th1 differentiation. Together these observations suggest that upregulation of Th2-genes may simultaneously drive a Th1 response, and that the opposing effects of these gene groups may regulate Th2-mediated eosinophil recruitment and allergic airway inflammation.

In the present study, RWE challenge upregulated the p47 GTPases Iigp and Tgtp. The exact function of these proteins is unknown. The subcellular location of Iigp has been identified as the Golgi apparatus and endoplasmic reticulum [14]. This suggests its possible role in processing of proteins or lipids traversing the ER, or more likely vesicular transport emanating from the ER. Tgtp is a T cell-specific GTPase whose expression is coupled to signals delivered through the T Cell Receptor. Tgtp is also induced in response to Ifng signaling, and confers protection against viral infection *in vitro* [31]. Elimination of different p47 GTPases in mice by gene targeting severely cripples the Ifng-regulated defense against *Toxoplasma gondii*, *Listeria monocytogenes*, *Mycobacterium spp*. and other pathogens [14]. The p47 GTPase Iigp regulates innate immunity and inflammation to murine *Chlamydia psittaci* infection [32]. The mechanism by which they eliminate these pathogens has been characterized. Iigp accumulates at vacuoles containing *T. gondii* via a GTP-dependent mechanism, and requires live parasites [33]. Vacuolar Iigp1 accumulations undergo a maturation-like process accompanied by vesiculation of the parasitophorous vacuole membrane [33]. This culminates in disruption of the parasitophorous vacuole, and finally of the parasite itself. However, the precise role of Iigp and Tgtp in allergic inflammation remains to be elucidated.

In the present study, the p65 GTPase Gbp1 was also upregulated by RWE challenge. Gbp1 is a family of p65 GTPases, and despite a high degree of sequence identity and the presence of an identical CaaX sequence with muGBP2, muGbp1 has a very homogeneous cytoplasmic distribution and fails to localize to intracellular vesicles [34], [35]. muGbp2 is induced in Ifn-treated macrophages and fibroblasts, and found in both a granular distribution throughout the cytoplasm and localized to vesicle populations of heterogeneous sizes [34], [35]. The localization of muGbp2 to vesicles is dependent on its isoprenylation. The different intracellular distributions of these two closely related family members suggests differential function(s). Studies have shown that Gbps are selectively up-regulated *in vitro* by a set of proinflammatory cytokines and Tlr agonists, as well as *in vivo* after *Listeria monocytogenes* and *Toxoplasma gondii* infection [14], [36]. After Ifngγ stimulation, Gbp1, 2, 3, 6, 7, and 9 are associated with intracellular *Toxoplasma* parasites; interestingly, virulent *Toxoplasma* interferes with mGbp recruitment [37]. Murine Gbp2 has been shown to inhibit replication of both vesicular stomatitis virus (VSV) and encephalomyocarditis virus (EMCV). The enzyme activity of p65 GTPase is not required for VSV inhibition, but is required for inhibition of EMCV, suggesting different mechanisms of inhibition for the two viruses [38]. Taken together, Gbps comprise an important set of host defense molecules. However, their precise role in allergic inflammation needs to be determined.

We demonstrated that RWE challenge upregulated Gadd45g by an Ifng-independent mechanism. Prior reports show that Gadd45g knockout mice are severely impaired in their capacity to activate p38 and JNK in response to TCR signaling, and produce much less Ifng upon re-stimulation, suggesting its role in the Th1 response [12], [39]. Thus upregulation of Gadd45g in response to allergen challenge could be important in counter-balancing extreme Th2 deviation.

Finally, our data also demonstrate that the Th1 genes remain upregulated longer than the Th2 genes. Taken together with the prolonged Th2 inflammation in Ifng-deficient mice, this suggests that persistent expression of these Th1-associated genes could be a physiological mechanism to resolve allergic inflammation after cessation of allergen exposure. Future studies conducted in allergen-sensitized mice with disruption of Gbp1, Iigp, Tgtp, Socs1, Cxcl9, Cxcl10 and Gadd45g genes will help dissect how these Ifng-dependent and -independent Th1-associated genes regulate allergic airway inflammation.
